# Epidemiological and microbiological characteristics of *S. aureus* pediatric infections in Colombia 2018–2021, a national multicenter study (Staphylored Colombia)

**DOI:** 10.3389/fped.2024.1386310

**Published:** 2024-06-04

**Authors:** Ivan Gutierrez-Tobar, Cristobal Carvajal, Pablo Vasquez-Hoyos, Alejandro Díaz-Díaz, Juan Pablo Londono Ruiz, Joam Andrade, Jhon Camacho-Cruz, Andrea Restrepo-Gouzy, Monica Trujillo-Honeysberg, Juan Gonzalo Mesa-Monsalve, Ignacio Perez, Richard Von Moltke, Maria Beltran-Echeverry, Jessica F. Toro, Angela P. Niño, Germán Camacho-Moreno, Juan Pablo Calle-Giraldo, Nancy Yhomara Cabeza, Lina Marcela Sandoval-Calle, Paola Perez Camacho, Jaime Patiño Niño, Paula Araque-Muñoz, Yazmin Rodríguez-Peña, Claudia Beltran-Arroyave, Yamile Chaucanez-Bastidas, Juan Lopez, Diego Galvis-Trujillo, Sandra Beltrán-Higuera, Ana-Cristina Marino, Natalia González Leal, Miguel Ángel Luengas Monroy, Derly Carolina Hernandez-Moreno, Rosalba Vivas Trochez, Carlos Garces, Eduardo López-Medina

**Affiliations:** ^1^ Department of Pediatrics, Clínica Infantil Santa Maria Del Lago, Bogotá, Colombia; ^2^ Department of Pediatrics, Clinica Infantil Colsubsidio, Bogotá, Colombia; ^3^ Staphylored LATAM, Bogotá, Colombia; ^4^ Universidad Finis Terrae, Santiago, Chile; ^5^ Department of Pediatrics, Fundacion Universitaria de Ciencias de la Salud (FUCS), Bogotá, Colombia; ^6^ Universidad Nacional de Colombia, Bogotá, Colombia; ^7^ Sociedad de Cirugía de Bogotá Hospital de San Jose, Bogotá, Colombia; ^8^ Department of Pediatrics, Hospital Pablo Tobon Uribe, Medellín, Colombia; ^9^ Department of Pediatrics, Hospital General de Medellín, Medellín, Colombia; ^10^ Department of Pediatrics, Hospital Militar Central, Bogotá, Colombia; ^11^ Department of Pediatrics, Fundación Universitaria Sanitas, Bogotá, Colombia; ^12^ Department of Pediatrics, Clínica Reina Sofia Pediátrica y Mujer, Bogotá, Colombia; ^13^ Clínica Las Americas, AUNA, Unidad de Enfermedades Infecciosas, Medellín, Colombia; ^14^ Universidad de los Andes, Santiago, Chile; ^15^ Department of Pediatrics, Clínica Medilaser, Neiva, Colombia; ^16^ Department of Pediatrics, Fundación Hospital de La Misericordia, Bogotá, Colombia; ^17^ Department of Pediatrics, Hospital Universitario Infantil de San José, Bogotá, Colombia; ^18^ Department of Pediatrics, Hospital San Juan de Dios, Armenia, Colombia; ^19^ Department of Pediatrics, Clinica Farallones, Cali, Colombia; ^20^ Department of Pediatrics, Clinica Versalles, Cali, Colombia; ^21^ Department of Pediatrics, Fundación Valle de Lili, Cali, Colombia; ^22^ Department of Pediatrics, Clinica Country, Bogotá, Colombia; ^23^ Department of Pediatrics, Clinica La Colina, Bogotá, Colombia; ^24^ Department of Pediatrics, Clinica El Rosario, Medellín, Colombia; ^25^ Department of Pediatrics, Hospital Infantil Los Ángeles, Pasto, Colombia; ^26^ Clinica Colsanitas, Bogotá, Colombia; ^27^ Department of Pediatrics, Clinica infantil Colsanitas, Bogotá, Colombia; ^28^ Independent Researcher, Manizales, Colombia; ^29^ Department of Pediatrics, Clínica Soma, Medellín, Colombia; ^30^ Department of Pediatrics, Hospital Universitario de San Vicente Fundación, Medellín, Colombia; ^31^ Department of Pediatrics, Clinica Cardiovid Medellín, Medellin, Colombia; ^32^ Centro de Estudios en Infectología Pediátrica, Cali, Colombia; ^33^ Universidad del Valle, Cali, Colombia; ^34^ Department of Pediatrics, Clínica Imbanaco, Cali, Colombia

**Keywords:** Colombia, LATAM countries, MSSA (methicillin-susceptible *Staphylococcus aureus*), MRSA—methicillin-resistant *Staphylococcus aureus*, pediatric infections, epidemiology, *S. aureus*

## Abstract

**Background:**

*Staphylococcus aureus* infections are a significant cause of morbidity and mortality in pediatric populations worldwide. The Staphylo Research Network conducted an extensive study on pediatric patients across Colombia from 2018 to 2021. The aim of this study was to describe the epidemiological and microbiological characteristics of *S. aureus* in this patient group.

**Methods:**

We analyzed *S. aureus* isolates from WHONET-reporting centers. An “event” was a positive culture isolation in a previously negative individual after 2 weeks. We studied center characteristics, age distribution, infection type, and antibiotic susceptibilities, comparing methicillin sensitive (MSSA) and resistant *S. aureus* (MRSA) isolates.

**Results:**

Isolates from 20 centers across 7 Colombian cities were included. Most centers (80%) served both adults and children, with 55% offering oncology services and 85% having a PICU. We registered 8,157 *S. aureus* culture isolations from 5,384 events (3,345 MSSA and 1,961 MRSA) in 4,821 patients, with a median age of 5 years. Blood (26.2%) and skin/soft tissue (18.6%) were the most common infection sources. Most isolates per event remained susceptible to oxacillin (63.2%), clindamycin (94.3%), and TMP-SMX (98.3%). MRSA prevalence varied by city (<0.001), with slightly higher rates observed in exclusively pediatric hospitals. In contrast, the MRSA rate was somewhat lower in centers with Antimicrobial Stewardship Program (ASP). MRSA was predominantly isolated from osteoarticular infections and multiple foci, while MSSA was more frequently associated with recurrent infections compared to MRSA.

**Conclusions:**

This is the largest study of pediatric *S. aureus* infections in Colombia. We found MSSA predominance, but resistance have important regional variations. *S. aureus* remains susceptible to other commonly used antibiotics such as TMP-SMX and clindamycin. Ongoing monitoring of *S. aureus* infections is vital for understanding their behavior in children. Prospective studies within the Staphylored LATAM are underway for a more comprehensive clinical and genetic characterization.

## Introduction

Pediatric infections caused by *Staphylococcus aureus* represent a significant global public health issue (1). These infections span a spectrum of severity, from mild skin and soft tissue infections to more serious conditions including disseminated disease with multiorgan failure and high mortality risk (2). Clinical studies suggest that the epidemiology, susceptibility profiles, and severity of *S. aureus* infections can vary according to population demographics and resistance patterns (3–5).

Regional differences exist in the prevalence of Methicillin-Sensitive (MSSA) and Methicillin-Resistant *Staphylococcus aureus* (MRSA) (3, 4, 6, 7). While MSSA predominates in certain European regions (6). In the United States, a meta-analysis of children and adults from studies spanning 1990 to 2012 found a range of MRSA prevalence in different regions, reaching a maximum of 83% (8) Other regional variations in susceptibility profiles to different antibiotics, such as clindamycin and TMP-SMX, have also been observed (9, 10). In addition, antibiotic susceptibility patterns have shown significant variations over time, with a surge in MRSA infections observed in the 2000s, and more recent reports indicate a resurgence of MSSA (3, 11, 12). Differential severity of infections and prognosis according to susceptibility patterns are conflicting. Infections caused by MRSA strains are often more severe, a trend that has been observed in Colombia and other regions (2, 13, 14).

The increasing prevalence of *Staphylococcus aureus* infections in pediatric populations raises significant concerns. A notable gap exists in local comprehensive, multicentric, and longitudinal studies that cover both MSSA and MRSA cases. This study is designed to determine the frequency and antibiotic susceptibility of pediatric *S. aureus* infections, as well as their epidemiological profiles. Furthermore, it aims to investigate regional variations in antibiotic resistance and identify temporal trends, including changes in the prevalence of resistance to various antibiotics among diverse *S. aureus* infections in different Colombian cities. By examining regional differences in infection severity and utilizing robust multicentric epidemiological data, this study seeks to provide a comprehensive overview of *Staphylococcus aureus* infections in the pediatric population. The findings are anticipated to have significant relevance both locally in Colombia and regionally, particularly in regions confronting similar challenges with *S. aureus* infections.

## Materials and methods

### Study design and patient selection

This was a retrospective observational study conducted from 2018 to 2021, including episodes of *S. aureus* isolations from either sterile (blood, pleural fluid, cerebrospinal fluid, peritoneal fluid, osteomuscular or articular fluid obtained during surgical procedures) or non-sterile sites (i.e., respiratory specimens, soft tissue abscesses) in patients under 18 years of age. Data were identified and gathered using the WHONET software, a widely used tool in Colombian hospitals developed by the World Health Organization for managing microbiology data (15).

### Data collection and management

Study data were collected and managed using REDCap electronic data capture tools hosted at Universidad Finis Terrae in Chile (16, 17). Hospital's participation workflow in REDCap started with email invitations to institutional referents. Each center then completed an online survey with institution characterization and uploaded four WHONET's export files (one per year). To ensure accuracy, written instructions, and a video tutorial on exporting data from WHONET were provided. A semi-automatic reactive workflow with data validations and email alerts was designed in REDCap to optimize time, security, privacy, and data quality. Online-local connections were supported through APIs (Application Programming Interfaces, which are sets of rules enabling software interactions).

### Data processing and standardization

The data processing involved a systematic approach: downloading REDCap and WHONET files for each center, followed by data cleansing and normalization into the study format using R/Python. This process included standardizing WHONET data's free texts (origin, specimen, and ward type) and converting Minimum Inhibitory Concentration (MIC) free text values into clinical interpretations (susceptible, intermediate, resistant), according to CLSI recommendations (18).

Data were aggregated at a patient-center level, and a 2-week interval was calculated for each event per patient per center. The refined WHONET data was then uploaded to a separate REDCap project for positive *S. aureus* culture results. Biomedical Informatics MDs collaborated with Pediatric Infectious Diseases specialists in iterative data engineering cycles. Tools used included two REDCap projects, Microsoft Excel, Google Sheets, R, and Python. An independent analysis team was employed to minimize bias.

### Definitions

A culture was defined as the identification of an *S. aureus* isolate, irrespective of its origin or if it was a repeated identification. Patients refers to individuals with at least one positive *S. aureus* culture. An event was defined as any instance with at least one positive *S. aureus* culture. Subsequent cultures within 14 days of the last positive culture were considered part of the same event, with each positive culture extending this window by an additional 14 days. A source was defined as the origin of body sites from which the isolate was identified. An event can have multiple sources, indicating that an isolate was identified from more than one body origin.

### Detailed information collection

All positive *S. aureus* cultures were included, and information on specimen type, culture origin, identification methods, resistance patterns, culture origins, and MIC values was obtained. Patient characteristics collected included age, institution type, capacity, services offered, bed numbers, geographical regions, cities, and other indicators.

### Ethical considerations

Participating centers obtained local ethical approval. The REDCap web platform was used for secure, anonymous data management, with decoding handled by the REDCap administrator at Finis Terrae University. This ensured data protection and security throughout the study.

### Statistical analysis

A non-probabilistic convenience sampling method was employed. We performed a descriptive analysis, presenting qualitative variables with absolute and relative frequencies, and quantitative variables with medians and IQR. Categorical variables were analyzed by case numbers and percentages. *χ*^2^ tests, following variance distribution analysis, were used to compare MSSA and MRSA, with statistical significance set at *p* < 0.05. All analyses were conducted using STATA® version 17.

## Results

Twenty centers from seven Colombian cities participated in the study. Most were private (17/20), cared for children and adults (16/20), had pediatric surgical services, pediatric intensive care unit (PICUs), and neonatal units. Sixteen centers performed molecular tests for *S. aureus* and resistance genes, and seventeen used therapeutic drug monitoring of vancomycin. Most had infectious disease departments, infection control programs, and Antimicrobial Stewardships Programs (ASPs), with variable distribution in the number of Infectious Disease specialists per bed per service (Table 1).

**Table 1:** Centers and resources for infectious diseases: key characteristics.

Centers (*n* = 20)	*n* (%) or medians [IQR]
Type of ownership	
Public	3 (15)
Private	17 (85)
Type of institution	
Non-academic	3 (15)
Academic	17 (85)
Type of center	
Children's hospital	4 (20)
General hospital	16 (80)
Area of influence	
Mainly urban population	19 (95)
Mainly rural population	1 (5)
Specialized medical services available^a^	
Pediatric intensive care unit (PICU)	17 (85)
Intermediate care unit	16 (80)
Neonatal unit	18 (90)
Pediatric surgery	20 (100)
Cardiovascular surgery	6 (30)
Oncology service	11 (55)
Transplants	3 (15)
Renal replacement therapy	7 (35)
Vancomycin level testing available	17 (85)
Molecular biology testing available	15 (75)
Automated microbiological identification systems	
VITEK 2 (BioMerieux)	13 (65)
BD Phoenix (BD diagnostics)	3 (15)
MicroscScan WalkAway (siemens health)	3 (15)
MALDI-TOF	1 (5)
Antimicrobial stewardship program	17 (85)
Infection prevention and control program	20 (100)
Bed-to-specialist ratio for pediatric infectious diseases.	
Pediatric beds	12 [9–27]
PICU beds	10 [8–25]
NICU beds	7 [5–17]
Pediatric infectious disease specialist onsite (days × week)	5 [5–5]
Number of pediatric infectious disease specialists on staff per center	
One	11 (55%)
Two	6 (30%)
Three	2 (10%)
Four	1 (5%)
Number of beds per hospital	
All beds	193 [154–413]
Pediatrics	67 [48–110]
PICU	11 [8–13]
NICU	18 [12–24]

*n*, sample size; %, percentage; med, median; IQR, interquartile range; PICU, pediatric intensive care unit; NICU, neonatal intensive care unit. VITEK 2 by BioMerieux; BD Phoenix by BD Diagnostics; MicroScan WalkAway by Siemens Health.

^a^ More than one choice per center.

**Figure 1:**
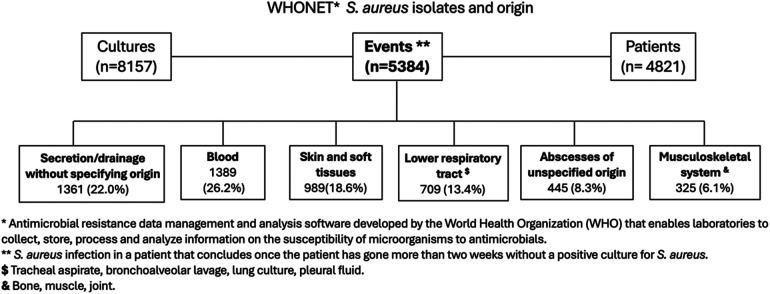
Source of *S. aureus* pediatric infections.

For the analysis, 5,384 events were included from 8,157 cultures of *S. aureus* that occurred in 4,821 patients (Figure 1). In the analyzed events, a total of 3,345 isolates of MSSA and 1,961 of MRSA were identified.

Blood was identified as the most frequent source, representing 1,389 (26.2%) events. Among these, 571 events (41.1%) were associated with an additional identified focus. Specifically, in 159 (27.8%), the associated focus was secretion or drainage of unidentified origin; in 119 (20.8%) events, the source was osteomuscular; and in 74 (12.9%), it originated from skin and soft tissues. Additionally, there were 989 (18.6%) events of skin and soft tissue infections and 709 (13.4%) events of lower respiratory tract infections.

Significant differences in MRSA frequency were evident across different cities (*p* < 0.001), with Neiva (53.8%) and Armenia (48.8%) reporting higher rates in contrast to Manizales (25.8%) and Medellín (30%). Additionally, a slight increase in the frequency of MSSA was noted among neonates compared to other age groups, as detailed in Table 2A.

**Table 2:** (**A**) Comparison of MSSA vs. MRSA event frequencies based on center characteristics and population. (**B**) Relevant clinical insights based on *S. aureus* susceptibility profile of events.

Variables	Total (*n*, %)	MSSA (3,345)^a^	MRSA (1,961)^a^	*p*-value
		*n* [%]		
Cities				**<0** **.** **001**
Bogota (EP^φ^: >11,100 k inhabitants)	1,995 (37.5)	1,211 [60.7]	784 [39.3]	
Medellin (EP^φ^: >4,000 k inhabitants)	1,502 (28.2)	1,052 [70.0]	450 [30.0]	
Cali (EP^φ^: >2,200 k inhabitants)	1,033 (19.4)	612 [59.2]	421 [40.8]	
Pasto (EP^φ^: >390 k inhabitants)	405 (7.6)	245 [61.9]	151 [38.1]	
Manizales (EP^φ^: >450 k inhabitants)	155 (2.9)	115 [74.2]	40 [25.8]	
Armenia (EP^φ^: >410 k inhabitants)	121 (2.2)	62 [51.2]	59 [48.8]	
Neiva (EP^φ^: >355 k inhabitants)	104 (1.9)	48 [46.1]	56 [53.8]	
Age				**0** **.** **052**
Neonates	598 (11.2)	408 [68.2]	190 [31.8]	
1–11 months	808 (15.2)	512 [63.4]	296 [36.6]	
1–5 years	1,210 (22.8)	738 [61.0]	472 [39.0]	
6–12 years	1,328 (25.0)	838 [63.1]	490 [36.9]	
13–18 years	1,362 (25.6)	849 [62.3]	513 [37.7]	
Type of ownership				**0** **.** **017**
Public	489 (9.2)	284 [58.1]	205 [41.9]	
Private	4,817 (90.8)	3,061 [63.5]	1,756 [36.5]	
Type of institution				0.151
Non-academic	342 (6.4)	228 [66.7]	114 [33.3]	
Academic	4,964 (93.6)	3,117 [62.8]	1,847 [37.2]	
Type of center				**0** **.** **008**
Children's hospital	1,825 (34.4)	1,106 [60.6]	719 [39.4]	
General hospital	3,481 (65.6)	2,239 [64.3]	1,242 [35.7]	
Area of influence				0.615
Mainly urban population	4,910 (92.5)	3,100 [63.1]	1,810 [36.9]	
Mainly rural population	396 (7.5)	245 [61.9]	151 [38.1]	
Specialized medical services available^b^				
Cardiovascular surgery				0.533
Available	3,076 (58.0)	1,950 [63.4]	1,126 [36.6]	
Non-available	2,230 (42.0)	1,395 [62.6]	835 [37.4]	
Oncology service				0.112
Available	4,095 (77.2)	2,605 [63.6]	1,490 [36.4]	
Non-available	1,211 (22.8)	740 [61.1]	471 [38.9]	
Transplant unit				**0** **.** **004**
Available	1,902 (35.8)	1,248 [65.6]	654 [34.4]	
Non-available	3,404 (64.2)	2,097 [61.6]	1,307 [38.4]	
Antimicrobial stewardship program				**<0** **.** **001**
Available	4,907 (92.5)	3,138 [63.9]	1,769 [36.1]	
Non-available	399 (7.5)	207 [51.9]	192 [48.1]	
Table 2B. Relevant Clinical Insights Based on *S. aureus* Susceptibility Profile of Events				
Number of events per patient				**<0** **.** **001**
One	4,746 (89.5)	2,945 [62.1]	1,801 [37.9]	
Two	320 (6.0)	199 [62.2]	121 [37.8]	
Three events or more	240 (4.5)	201 [83.8]	39 [16.2]	
Number of body sources of positive cultures per event				**<0.001**
Single source	4,680 (88.3)	3,004 [64.2]	1,676 [35.8]	
Two sources	489 (9.2)	280 [57.3]	209 [42.7]	
Three or more sources	133 (2.5)	58 [44.6]	75 [56.4]	
Most frequent culture sources^c^				
Blood	1,389 (26.2)	871 [62.7]	518 [37.3]	0.763
No	3,917 (73.8)	2,474 [63.2]	1,443 [36.8]	
Skin and soft tissues	989 (18.6)	606 [61.3]	383 [38.7]	0.202
No	4,317 (81.4)	2,739 [63.4]	1,578 [36.6]	
Lower respiratory	709 (13.4)	477 [67.3]	232 [32.7]	**0** **.** **012**
No	4,597 (86.6)	2,868 [62.4]	1,729 [37.6]	
Musculoskeletal system	325 (6.1)	174 [53.5]	151 [46.5]	**<0** **.** **001**
No	4,981 (93.9)	3,171 [63.7]	1,810 [36.3]	
Vascular devices and accesses	134 (2.5)	92 [68.7]	42 [31.3]	0.173
No	5,172 (97.5)	3,253 [62.9]	1,919 [37.1]	
Clinically significant isolates and associated number of sources				
Osteoarticular infection (*N* = 325)				**0** **.** **007**
Single	144 (44.1)	79 [54.9]	65 [45.1]	
Two additional sources	108 (33.2)	67 [62.0]	41 [38.0]	
Three or more additional sources	73 (22.5)	28 [38.4]	45 [61.4]	
With bacteremia	119 (36.6)	51 [42.9]	68 [57.1]	**0** **.** **003**
No	206 (63.4)	123 [59.7]	83 [40.3]	
Lung/lower respiratory infection (*N* = 709)				**<0.001**
Single^§^	605 (85.3)	424 [70.1]	181 [29.9]	
Two additional sources	78 (11.0)	43 [55.1]	35 [44.9]	
Three or more additional sources	26 (3.7)	10 [38.5]	16 [61.5]	
With bacteremia	72 (10.2)	35 [48.6]	37 [51.4]	**<0.001**
No	637 (89.8)	442 [69.4]	195 [30.6]	
Skin and soft tissues (*N* = 798)				**0** **.** **004**
Single^§^	853 (86.3)	539 [63.2]	314 [36.8]	** **
Two additional sources	91 (9.2)	48 [52.7]	43 [47.3]	** **
Three or more additional sources	45 (4.5)	19 [42.2]	27 [57.8]	** **
With bacteremia	74	30 [40.5]	44 [59.5]	**<0.001**
No	915	576 [63.0]	339 [37.0]	** **
Bacteremia (*N* = 1,389)				**<0.001**
Single^§^	952 (68.5)	639 [67.1]	313 [32.9]	** **
Two additional sources	316 (22.8)	182 [57.6]	134 [42.4]	** **
Three or more additional sources	121 (8.7)	50 [41.3]	71 [58.7]	** **
Antibiotic susceptibility				
Clindamycin (*N* = 4,845)				**<0.001**
Susceptible	4,570 (94.3)	2,938 [64.3]	1,632 [35.7]	** **
Intermediate/resistant	275 (5.7)	126 [45.8]	149 [54.2]	** **
TMP/SMX (*N* = 4,964)				**<0.001**
Susceptible	4,877 (98.3)	3,161 [64.8]	1,716 [35.2]	
Intermediate/resistant	87 (1.7)	19 [21.8]	68 [78.2]	

MSSA, methicillin-sensitive *Staphylococcus aureus*; MRSA, methicillin-resistant *Staphylococcus aureus*; *n*, sample size; (Col %), col frequency as a percentage; [Row %], row frequency as a percentage.

*p*-value was calculated using the *χ*^2^ test.

Bold values indicate statistically significant results for a cut-off point of less than 0.05.

^a^ In 78 events, it was not possible to determine MSSA or MRSA.

^b^ More than one choice per center.

^c^ The same event can have multiple positive origins.

^§^ Single: Indicates that the isolate was exclusively found in the body site mentioned in the header.

^ϕ^ EP: estimated population as of 2021.

The median age of the patients was 5 years, with a min-max age range of 1–12 years. The age group with the highest prevalence included adolescents aged 13–18, accounting for 1,362 (25.6%) events, closely followed by the 6–12 years age group with 1,328 (25.0%) events.

The prevalence of MSSA compared to MRSA varied across different healthcare settings. In private centers, MSSA was more prevalent than in public centers (63.5% vs. 58.1%, *p* = 0.017). A similar pattern was observed when comparing general hospitals to pediatric hospitals (64.3% vs. 60.6%, *p* = 0.008), and in centers with transplant units (65.6% vs. 61.6%, *p* = 0.004). The presence of Antimicrobial Stewardship Programs (ASP) correlated with a higher frequency of MSSA compared to centers without ASP (63.9% vs. 51.9%, *p* < 0.001). However, the prevalence of MSSA vs. MRSA did not significantly differ based on urban or rural care settings or university affiliation, as detailed in Table 2A.

The frequency of MSSA vs. MRSA was similar in patients with single events (62.1% vs. 37.9%) and those with two events (62.2% vs. 37.8%). However, the presence of three events or more was associated with a higher frequency of MSSA (83.8% vs. 16.2%, *p* < 0.001). In contrast, MRSA was more frequent if three or more body sources had a positive culture during the same event (56.4 vs. 44.6%, *p* < 0.001).

While MSSA was more commonly detected across various culture sources, the identification of a pulmonary origin was significantly more associated with MSSA (*p* = 0.012). In contrast, musculoskeletal origins had a higher association with MRSA (*p* < 0.001) compared to other infection sites. Regardless of the body source of the infection, as the number of identified additional sources increased to three or more, the relationship inverted, resulting in a higher frequency of MRSA identification. Similarly, the presence of bacteremia was more frequently associated with MRSA. Regarding antibiotic susceptibility, clindamycin and TMP/SMX demonstrated high susceptibility against *S. aureus*, irrespective of the resistance profile. Nevertheless, MRSA isolates demonstrated a significantly higher resistance level when compared to MSSA isolates. Specifically, resistance to clindamycin was documented at 4.1% in MSSA, which increased to 8.4% in MRSA isolates. In a similar trend, resistance rates for TMP-SMX rose from 0.6% in MSSA to 3.8% in MRSA (*p* < 0.001), Table 2B.

**Figure 2:**
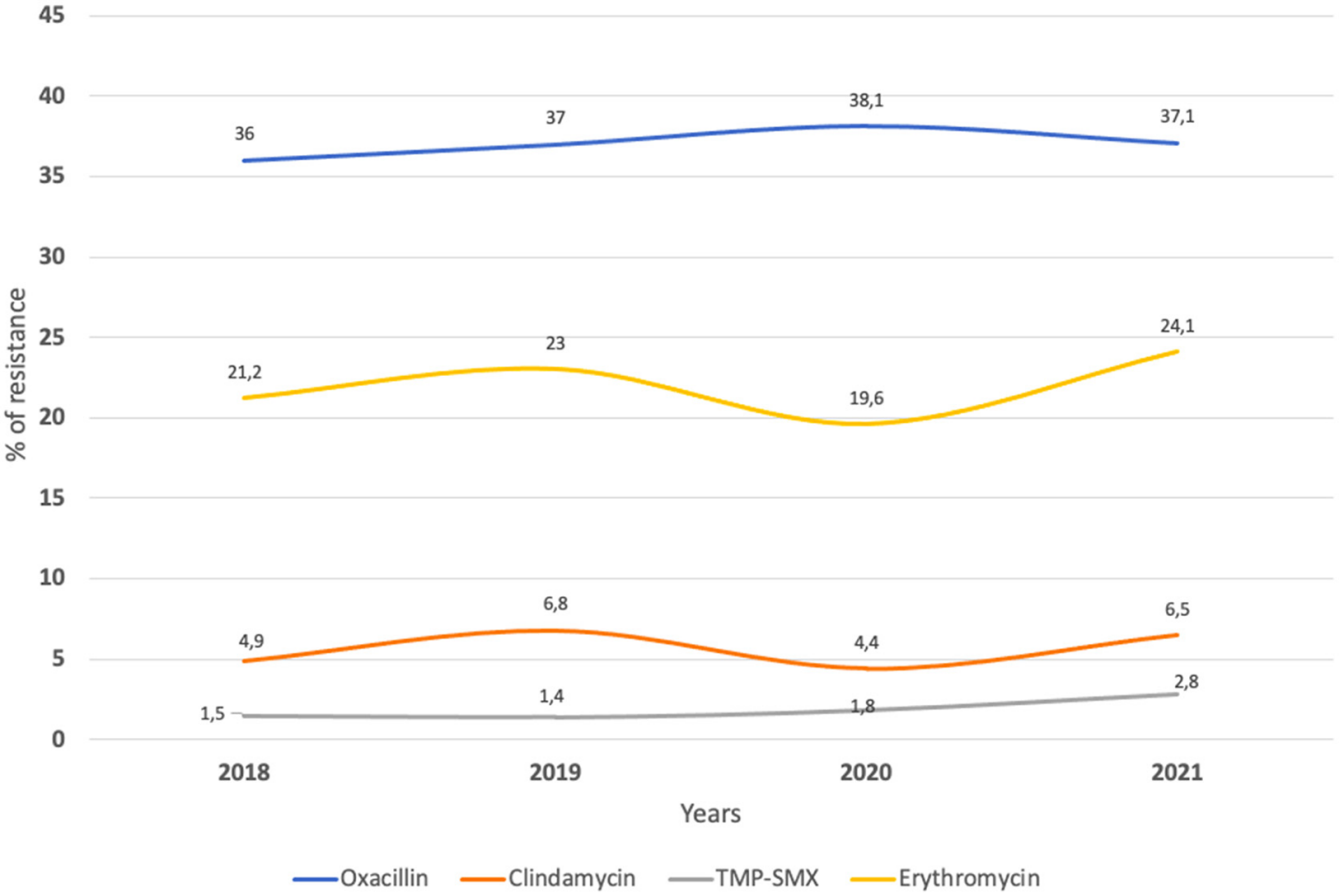
Percentage of resistance to different antibiotics 2018–2021 for single events (*n *= 5,384). TMP-SMX, trimethoprim-sulfamethoxazole.

Variations in antibiotic susceptibility among *Staphylococcus aureus* isolates were evident across different cities, as detailed in Supplementary Figure S1. No significant variations in antibiotic susceptibility were observed throughout the duration of the study, as illustrated in Figure 2.

## Discussion

This multicentric study conducted across Colombia provides an analysis of the microbiological profile of *S. aureus* infections in the pediatric population. Analysis of positive culture reports revealed a predominance of MSSA among the subjects. However, regional, and center-specific variations were noted in antibiotic resistance patterns, with generally high levels of susceptibility to clindamycin and TMP-SMX. Notably, MRSA was associated with more severe clinical presentations. This was evidenced by an increased frequency of MRSA as the number of body sources per event rose (multifocal infections), and in all cases of bacteremia associated with another focus. Conversely, MSSA was more frequently encountered in patients with recurrent infections.

### Differences in prevalence between MSSA and MRSA by location and type of centers

We found significant differences in MSSA and MRSA prevalence, and resistance patterns to other antibiotics among different cities in Colombia. Regional variations in the resistance of *S. aureus* by region have been previously described, with elevated MRSA rates observed in southern and eastern Europe compared to northern Europe (6). Marked regional differences have also been reported in Asia (19). In addition, higher colonization rates of MRSA have been observed in Adult Intensive Care Units (ICUs) located in the southern latitudes of the United States (20). The underlying causes for the variations in resistance patterns have not been fully identified. However, factors such as inadequate infection control measures and increased antibiotic usage are recognized as contributing to the higher prevalence of MRSA (7); although a Latin American study did not establish a clear relationship between antibiotic consumption and MRSA frequency (21) this could still be a factor associated with *S. aureus* susceptibility patterns. The impact of per capita income on MRSA prevalence may also have some relevance (22). Characteristics of the cities in this study, potentially influenced by patient demographics, infection control strategies, and weather among others, may contribute to these differences. Identifying the presence of different regional resistance patterns could contribute to the development of treatment recommendations for *S. aureus* infections tailored to specific regions, based on local data and epidemiology. These aspects could be explored in future research, and it would be valuable to analyze this phenomenon and potential regional variations as part of future phases of the Staphylored LATAM study.

Children's hospitals showed a higher frequency of MRSA prevalence compared to general hospitals (39.4% vs. 35.7%, *p* < 0.008). Similarly A Chicago study during the 2006 US MRSA peak reported a higher frequency of MRSA in children compared to adults (72.1% vs. 64.3%) showing greater resistance to non-beta-lactam antibiotics (23). In contrast, a Korean study found higher resistance in adults compared to children to clindamycin and ciprofloxacin, indicating that factors influencing resistance patterns vary across age groups and extend beyond oxacillin (24).

On the other hand, the availability of certain services, such as transplantation, was associated with lower frequency of MRSA, while this was not seen for other units like oncology and cardiovascular surgery. McNeil et al. at Texas Children's Hospital in the United States, identified 41 *S. aureus* infections in solid organ transplantation patients, with a similar percentage of MRSA and MSSA infections (47.5% and 52.5%) (25). The same group had previously described a predominance of MSSA infections in pediatric oncology patients (26).

Unique factors in children, such as different environments, daycare and school attendance, specific behaviors, and distinct antibiotic use, may influence these differences in antibiotic resistance. Similarly, centers with transplant units, despite potentially higher antibiotic consumption, often maintain higher standards of care and employ strategies for cross-transmission prevention, which could contribute to a decrease in MRSA frequency. Further research is imperative to unravel the contributing factors to the heightened MRSA prevalence in certain pediatric populations and possibly to develop targeted strategies for infection control and antibiotic stewardship in diverse healthcare environments. These findings underscore the complex dynamics of MRSA prevalence throughout time in different hospital settings.

We found a lower frequency of MRSA in institutions with established Antimicrobial Stewardship Programs (ASPs) (27, 28), highlighting the potential role these programs may play in not only reducing the frequency of MRSA but also potentially curbing the transmission of MRSA and other resistant bacteria (29). Interestingly, a lower frequency of MSSA was also noted in private institutions. These findings reinforce the hypothesis that dedicated strategies and resources for infection prevention and the rational use of antibiotics appear to impact MRSA frequencies. This should encourage efforts to optimize such programs. Investment in resources to enhance ASPs could also influence the patterns of resistance, including MRSA. The correlation between the presence of ASPs and lower MRSA rates underscores the need for robust infection control practices and policies, especially in managing antibiotic resistance, which remains a global healthcare challenge.

### Trends in oxacillin resistance

In our study, we observed an overall oxacillin resistance rate of 36%, with minimal variation across different years. Similar trends of non-significant variations over time have been reported in our country. According to data from the Bacterial Resistance Control Group (GREBO) in Bogota, the MRSA rate in pediatric settings ranged from 34.3% to 46.1% in 2011 (26), 34.8%–42.1% in 2015 (30) and increased to 46.2%–49.2% in 2021 (31). Montes et al. also found MRSA rates between 40% and 50% in pediatric isolates from 2009 to 2017, which aligns with the stable 45% MRSA rates in adults reported by Castro-Orozco et al. during a similar time period (32, 33).

The epidemiology of community and hospital-acquired MRSA has undergone significant changes. Initially more common in hospitals, MRSA became widespread in the late 1990s and early 2000s, driven by clonal types like the USA300 clone, resulting in over 60% oxacillin resistance in certain populations (11). However, recent studies have observed a decline in methicillin resistance in both pediatric and adult infections (3, 10). Khamash et al. reported a significant decrease in methicillin resistance in community-onset (48%–15%; *p* < 0.01) and hospital-onset (32%–13%; *p* < 0.01) infections (9), in another US pediatric study, oxacillin susceptibility declined from 59.4% in 2005 to a low point of 53.6% in 2007, but steadily increased to 68% by 2014 (12). Similarly, Hulten K et al. demonstrated changes in community-acquired *S. aureus* infections in a large pediatric hospital in Texas from 2007 to 2014. The prevalence of CA-MRSA infections, primarily affecting the skin and soft tissue, decreased by 60%, while MSSA remained stable over time and accounted for 66% of invasive infections by 2014 (33, 34).

Recent data has shown stable trends in oxacillin susceptibility among pediatric community-onset MRSA infections in the US. In a multicenter retrospective study conducted between 2015 and 2020, the overall oxacillin susceptibility rate was 67%, with no significant changes over time (71.5% in 2015 and 70.8% in 2020, *p* = 0.71). Overall, changes in MRSA and MSSA epidemiology can vary by region and over time. While some regions have seen a decline in MRSA infections, others have identified specific clones that have predominated. This can be influenced by a combination of factors such as infection prevention strategies (which have been effective in reducing hospital onset infections and transmission), changes in treatment principles, variations in virulence and genetic diversity, and differences in the types of infections captured (invasive vs. non-invasive, or community vs. hospital-acquired) (4, 9, 34, 35). Despite the prevalence of MSSA, the reasons behind the lack of a notable decline in MRSA frequency—a finding that contradicts existing literature—within the studied cities remain unclear. Furthermore, since our study did not identify any distinction between community-acquired (CA) and hospital-acquired (HA) infections, we cannot ascertain any differential behavior based on their origin. It is crucial to maintain surveillance in order to determine whether, as observed in other regions, there will be a greater and more progressive increase in MSSA prevalence in the coming years.

### Other antibiotics and their trends in susceptibility over time

*S. aureus* had a high susceptibility (>90%) to clindamycin overtime. This pattern has previously been reported in the pediatric population in Colombia (31). We also found that the susceptibility to clindamycin in the MSSA group is significantly higher than in the MRSA group (95.9% vs. 91.6%). However, both groups maintained a susceptibility >90%, which differs from what has been reported in a pediatric US population, in whom the sensitivity to clindamycin was 75% (36). These variations in susceptibility patterns may be due to various contributing factors. The first factor is the circulation of the ST8-MRSA-IVc clone (USA300-LV) in our country, which has been associated with this susceptibility pattern (36). The second factor could be the limited availability of pediatric formulations of clindamycin in our country, leading to its reduced usage and potentially exerting less selective pressure on the development of resistance to this antibiotic. We plan to conduct phylogenetic studies within the Staphyred to further understand the nature of these differences.

These findings support the use of clindamycin as an adequate empiric treatment option for *S. aureus* infections in the pediatric population, especially for osteoarticular infections, and skin and soft tissue infections, as recommended by national and international guidelines (37, 38). TMP-SMX was also highly susceptible in both MSSA and MRSA isolates. These findings also differ from previous national and US antimicrobial resistance reports indicating increase in the resistance to TMP-SMX by *S. aureus* (9, 31) Locally, TMS-SMX is currently recommended for skin and soft tissue infections (37). Additionally, TMP-SMX could also be an option for consolidation therapy in pediatric osteoarticular infections, especially in our setting, where clindamycin oral suspension is limited or not available.

### Clinical differences between MRSA vs. MSSA

We were able to explore some clinical differences between MRSA and MSSA. The most remarkable finding was that MRSA was associated with a greater number of infection foci within the same event, particularly in the setting of bacteremia, where we observed a higher frequency of MRSA regardless of the infection focus. A previous study conducted in Bogotá, Colombia, reported that MRSA bacteremia was more frequent than MSSA when the associated focus was osteoarticular, showing prevalence rates of 73% compared to 53%. Meanwhile, the same study identified that MSSA was more prevalent in bacteremias associated with soft tissue infections, with a prevalence rate of 21% compared to 6% for MRSA (2). Another study comparing MSSA vs. MRSA in children did not find a difference in the frequency of bacteremia (39). From a clinical perspective, these findings may suggest potential treatment approaches, as the presence of multifocal disease and associated bacteremia appears to be more frequently associated with MRSA. Consequently, in specific cases and considering local epidemiology, this type of clinical presentation could guide empirical antibiotic management.

We found that as the number of events per patient increased (recurrence), the frequency of MSSA also increased. Different studies have provided variable findings regarding recurrence and *S. aureus* resistance. Bae et al. in South Korea, revealed a significant association between MRSA infections and higher recurrence rates (70.6% vs. 50.3%, *p* < 0.001) (13). Similarly, Bocchini et al. from Texas Children's Hospital reported an increased likelihood of subsequent MRSA infections compared to MSSA infections (*p* < 0.001) (36). In contrast, Michelle S. Hsiang et al. did not find any difference in *S. aureus* resistance profile and recurrence frequency (39). On the other hand, studies focusing on recurrent skin and soft tissue infections, more frequently identified MSSA as the primary causative agent in recurrences, especially in cases with the presence of PVL-positive strains (40, 41). Notably, a particularly high frequency of PVL has been described in MSSA in Colombia (40). Although we don't have data on the source of recurrences, since skin and soft tissues were one of the most frequent isolations, it could be hypothesized that this phenomenon is related to a higher frequency of infections from this source. This information is worth analyzing in more detail in later phases of Staphylored.

Altogether, our findings suggest a link between recurrence and severity according to resistance patterns. However, this study only considered WHONET-reported information, excluding clinical determinants like comorbidities and infection origin. A future Staphylored LATAM, Colombia project will prospectively evaluate these factors. Further research is also needed to explore associations between severe *S. aureus* isolates, recurrence, and virulence mechanisms like PVL and enterotoxins, often observed in MRSA (35).

### Limitations

This retrospective study has several limitations, including the lack of clinical data, patient outcomes, and risk factors. It relies on automated microbiology systems, which may have errors in sample origin and labeling. This issue is particularly significant for the inability to determine the exact origin in cases of “abscess” or “drainage or secretion”. Interpretation of recurrence analysis should be cautious, as new cases at different institutions may not be accurately classified as recurrences. Primary blood isolates, though highlighted, are infrequent in pediatric *S. aureus* infections and may represent primary foci where no cultures were obtained. Detailed blood-related analysis is needed. Institutional and city data may not represent individual behavior of *S. aureus* infections in Colombian pediatric populations. Given the retrospective nature of the study and the fact that the information was obtained from cultures without a review of the clinical record, it is not possible to determine whether cultures from non-sterile sites represent colonization or infection. However, considering that only inpatient cultures were selected, this possibility might be reduced. Despite these limitations, our work is groundbreaking for our region and lays the foundation for prospective clinical collaborations.

## Conclusions

This multicenter study, across Colombia, reveals a predominance of MSSA isolates, with some variations observed among centers and regions. The prevalence of MRSA appears to remain stable over time. MRSA is more frequently observed in cases of higher severity, such as multiple infections, bacteremia, and osteoarticular infections, while MSSA is more common in recurrent cases. TMP-SMX and clindamycin demonstrate excellent sensitivity in different regions and serve as effective alternatives in non-severe cases or step-down therapy. Ongoing monitoring of *S. aureus* infections in pediatric settings, including epidemiological, microbiological, and clinical characteristics, is necessary. The next phase of the Staphylored LATAM study will continue to assess the clinical characteristics, progression, and outcomes of *S. aureus* infections in pediatric patients across Colombia and potentially in other regions of LATAM. Including genotyping analysis in this phase aims to improve our understanding of these infections, which could contribute to more effective management approaches in pediatric care.

## Data Availability

The data analyzed in this study is subject to the following licenses/restrictions: The datasets can be shared if the editors consider it, however, they cannot be publicly shared because they correspond to data from each participating institution. Requests to access these datasets should be directed to IG-T, ivanfelipegutierrezt@gmail.com.
