# Supplementary material for: Epidemiological and microbiological characteristics of S. aureus pediatric infections in Colombia 2018–2021, a national multicenter study (Staphylored Colombia)
Source: Front Pediatr. 2024 Jun 4;12:1386310. doi: 10.3389/fped.2024.1386310 (PMC11183781; doi:10.3389/fped.2024.1386310)
Supplement: Supplementary file 1 [file Image1.pdf]

Supplementary Figure 1. Antibiotic Resistance Frequency by City

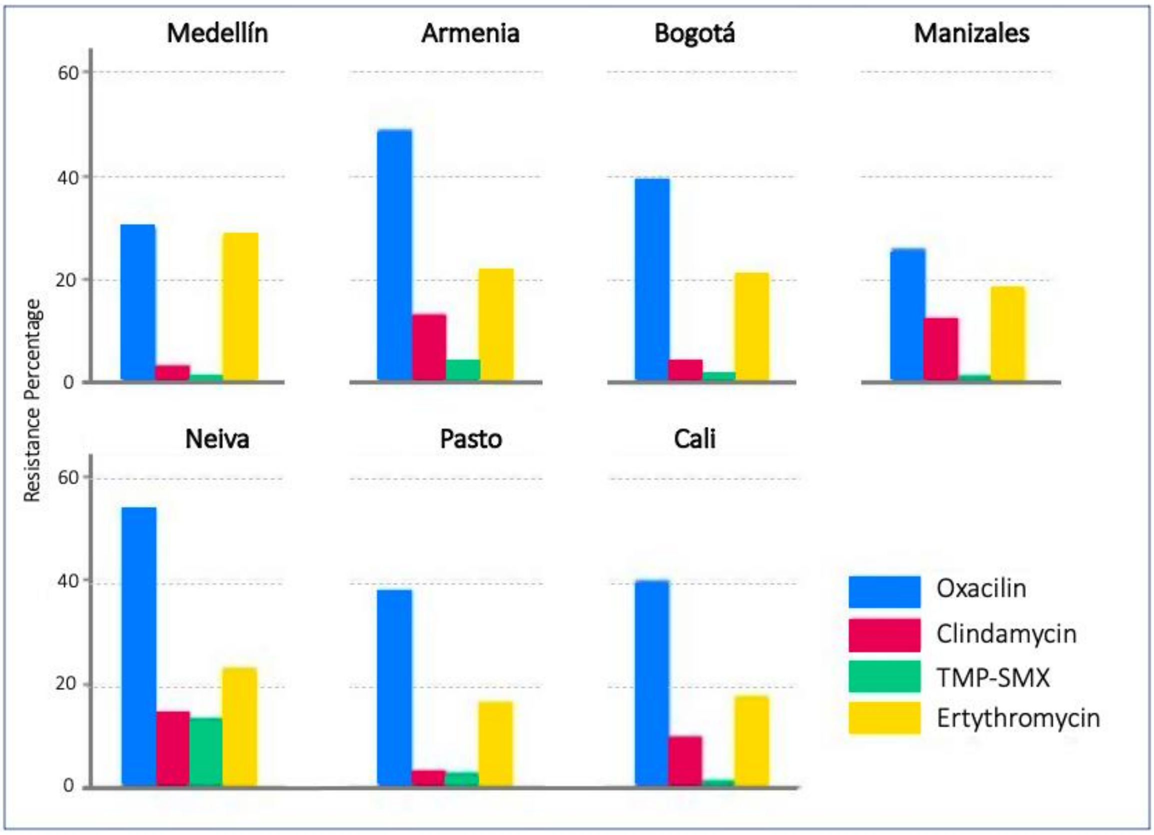

TMP-SMX: Trimethoprim-Sulfamethoxazole
